# Comparison of Lasers and Desensitizing Agents in Dentinal Hypersensitivity Therapy

**DOI:** 10.3390/dj11030063

**Published:** 2023-02-27

**Authors:** Francesca Cattoni, Lucrezia Ferrante, Sara Mandile, Giulia Tetè, Elisabetta Maria Polizzi, Giorgio Gastaldi

**Affiliations:** Department of Dentistry, IRCCS San Raffaele Hospital, Dental School, Vita-Salute San Raffaele University, 20132 Milan, Italy

**Keywords:** hypersensitivity and diode laser, hypersensitivity and Nd-Yag laser, dentin exposure, sensitive dentine

## Abstract

The main objective of this review is to verify the validity of laser therapy in the treatment of dentin hypersensitivity, an extremely common problem in patients, with Nd: YAG lasers or high- and/or low-power diode lasers to obtain a definitive protocol for the treatment of hypersensitivity, given the multiplicity of laser treatments proposed by the numerous authors evaluated. The authors performed an electronic search on PubMed, favouring it as a search engine. Lasers represent a means of treating dentin hypersensitivity, used alone and/or in conjunction with specific products for the treatment of such a pathology. The selected articles that examined diode lasers were divided according to the wattage (w) used: low-level laser therapy protocols, i.e., those using a wattage of less than 1 W, and high-level laser therapy protocols, i.e., those using a wattage of 1 W or more. Regarding the Nd: YAG laser, it was not necessary to subdivide the studies in this way, as they used a wattage of 1 W or more. A total of 21 articles were included in the final selection. Laser therapy was found to be effective in the treatment of dentin hypersensitivity. However, the level of effectiveness depends on the laser used. The results obtained from this review show that both the Nd: YAG laser and the diode laser (high and low power) are effective in the treatment of dentin hypersensitivity. However, the high-power laser appears to be more effective in combination with fluoride varnish and the Nd: YAG laser achieved greater long-term benefits than the diode laser.

## 1. Introduction

Dentine is very sensitive to touch, heat, cold, sweet food, etc., as nerve fibres are found almost everywhere. Nerve fibres are found in the peri odontoblastic space of the predentin and in most mineralised dentin, on the pulpal side. The hydrodynamic theory of pain induction is based on the movements of the dentin tubule contents, which stimulate nerve endings in the odontoblast layer. Another theory is based on the possible conduction property of the pain stimulus by the odontoblasts themselves. Indeed, it has been suggested that, in certain animals, odontoblasts may originate from the neural crest cells: it would therefore be possible that they also possess properties that enable them to transmit sensory stimuli. In this respect, the serrating or communicating junctions (gap junctions) between the various cellular elements of the odontoblastic and subodontoblastic regions may be particularly important [1].

These junctions allow the exchange of fluids and ions and have low electrical resistance, which allows action potentials to diffuse without delay. The pain that originates from dentin is sharp, stabbing, and short-lasting, and would be typical of A-fibre activity. Dentinal hypersensitivity is a condition characterized by the appearance of short, intense pain that persists until the stimulus is removed, and is not related to any dental pathology other than the anatomical exposure of the cervical dentine to the oral environment, leading to the exposure of the open dentinal tubules and the reactivity of the dental pulp nerve to the external environmental stimuli. Dentin hypersensitivity is a treatable condition, and its management involves, as a first step, a differential diagnosis to ascertain that the dental pain is not related to other problems and pathologies. As far as aetiology is concerned, Brännstrom’s hydrodynamic theory is the most accredited in the aetiopathogenesis of dentin hypersensitivity [2].

The evocative stimulus (e.g., cold) can cause a rapid displacement of pulpal fluid within the dentinal tubule system (contraction, expansion, displacement). The direction of this flow can be centrifugal (i.e., from the pulp outwards) or centripetal (i.e., from the oral environment towards the pulp). The displacement of pulpal fluid induces changes in the shape of receptors (mechanoreceptors) that accompany the odontoblast extension inside the dentinal tubule for a short distance. These receptors are said to be able to transduce the ‘deformation’ signal into a pain nerve impulse that is conducted to the pulpal nerve plexus (Raschow’s plexus) via myelin fibres with a high conduction velocity called A-delta ‘Aδ’ fibres. Stimulation of the ‘Aδ’ fibres appears to be correlated with acute pain symptoms of odontogenic origin, including dentinal hypersensitivity. Hypersensitivity is due to the opening of the dentinal tubules (very small and very numerous tunnels that connect the outside with the inside of the tooth), making it possible for stimuli to pass through to irritate the dental pulp. In particular, thermal lowering, dehydration obtained by means of an air jet, evaporation, and the application of an osmotic stimulus (sugar, acid, salt, etc.) induce the centrifugal displacement of the tubular fluid and are able to activate the nerve endings more effectively than tactile and thermal stimuli of the temperature increase that produce the displacement of the fluid in the direction of the pulp [3]. It has been said that for the painful manifestation of dentin hypersensitivity to occur, it is necessary to have the dentin exposed to the evocative stimuli of the oral environment. Painful symptoms only appear if there is a flow of pulpal fluid through the dentine and it is therefore necessary for the two ends of the dentinal tubule to be pervious both to the pulpal tissues and to the oral environment; therefore, the concept that must be considered is that of dentinal permeability. The diagnosis of dentinal hypersensitivity requires an accurate anamnestic, dental, and radiological examination. A differential diagnosis is necessary to exclude existing carious pathologies, pulpal pathologies, current prosthetic, or conservative therapies that may be the cause, and it is also necessary to investigate each patient’s vicious habits. Hypersensitivity testing should form part of the initial objective examination and be performed by a direct air jet on the dental elements to be evaluated [4].

Physical, chemical, pathological, biological, and/or developmental problems that result in dental and/or periodontal damage or defects cause dentin exposure. Various clinical conditions believed to play a role in the development of dentin hypersensitivity include erosion, abrasion, corrosion, and enamel abrasion [5]. Dentin hypersensitivity is one of the most common causes of discomfort among patients [6].

In patients with dentinal hypersensitivity, the affected teeth become sensitive to generally harmless environmental stimuli. Cold, heat, chemicals (acidic or sweet fruit, food, drinks, etc.), and airflow can induce a short, sharp pain that may impair daily activities such as eating, drinking, talking, and brushing teeth. This severe disorder can last more than 6 months and become a constant nuisance, attacking the emotional and psychological sphere of the individual [7,8,9,10].

Dental hypersensitivity can be a result of:

− Gingival recession, an important predisposing factor as it exposes the cervical dentine and the root
− Aging
− Dehiscence of soft tissue
− Brushing that is too aggressive

These issues often lead to the apical displacement of the gingival margins, resulting in exposure of the dentine, which can then lead to dentinal hypersensitivity [11,12,13,14]. There is still no single treatment for dentin hypersensitivity, and the market offers such a wide range of products that orientation around them appears complex. Various substances and even machines such as lasers can be used to treat dentin hypersensitivity [10]. The proposed products are divided into chemical agents (e.g., potassium salts, fluoride, sodium citrate, corticosteroids, silver nitrate, strontium chloride, formaldehyde, and calcium hydroxide) and physical agents (composites, microfilled and unfilled resins, sealants, dentin adhesives, glass ionomer cements, etc.) [15,16,17].

The specific machines for treating dentin hypersensitivity are lasers. Protocols that can be defined as specific for such treatment are low and medium power and have been reported to be effective in hypersensitivity treatment. These machines can act either by reducing pulpal nerve excitability or by inducing the occlusion of the dentinal tubules [18,19]. Laser therapy, however, is costlier than the therapies commonly used with the various desensitizing agents. Therefore, to ‘justify’ the expense to the patient, it is important that the doctor is able to demonstrate the validity of this treatment and guarantee, as far as possible, that this treatment offers more benefits than desensitizing agents and is, above all, longer lasting. The main objective of this review is to verify the validity of laser therapy with Nd: YAG laser or high- and/or low-power diode laser to arrive at a definitive protocol for the treatment of hypersensitivity.

## 2. Materials and Methods

### Search Strategy and Selection Criteria

The authors performed an electronic search on PubMed by entering as keywords ‘laser’ OR ‘dentin hypersensitivity’ OR ‘Nd: YAG’ OR ‘diode laser’ OR ‘desensitizing’ OR ‘non-carious cervical lesion’. The search field was narrowed to select only studies performed from 2003 to 2020. The search was limited to human subject studies and studies that met other eligibility criteria.

The inclusion criteria were:

- Randomized clinical trials
- Controlled studies
- Double-blind controlled studies
- Studies with split-mouth protocol
- Studies with a follow-up of at least 3 months
- Studies with a minimum of seven patients of both sexes, aged between 20 and 60 years.
- Studies with Nd: YAG laser
- Studies with diode lasers
- Studies comparing the Nd: YAG laser with the diode laser

The exclusion criteria were:

- In vitro studies
- Animal studies

The selected articles that examined diode lasers were divided according to the power (w) used: studies in which low-level laser therapy, or protocols that use wattage lower than 1 W, was used and studies in which high-level laser therapy, or protocols that use wattage equal to or greater than 1 W, was used. As for the Nd: YAG laser, there was no need to make this division as all the studies used wattage equal to or greater than 1 W.

## 3. Results

### 3.1. Selection of Studies

**Figure 1:**
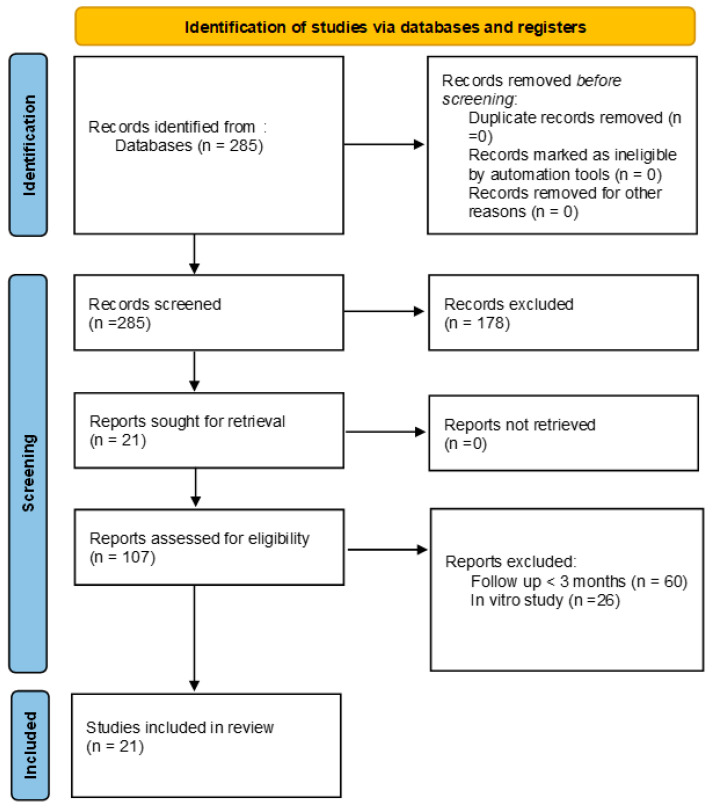
Study selection process and literature search results (PRISMA flowchart).

After research, we identified 285 articles. After examining their abstracts, we removed 127 studies because the topic was not relevant to the objective of this review and excluded another 51 studies because they were not available in full text. Another 86 articles were excluded because they did not meet the inclusion criteria. We finally had 21 articles: six articles that study the effectiveness of the Nd: YAG laser and compare and/or associate it with different sensitizers, such as Gluma, MI Varnish, and sodium fluoride; five articles that study the effectiveness of the low-power diode laser and compare and/or associate it with different desensitizers, such as 8% calcium carbonate, Gluma, fluorine, and Oxa Gel; five articles that study the effectiveness of the high-power diode laser and compare and/or associate it with various desensitizers, such as sodium fluoride, 5–10% potassium nitrate gel, and Gluma; and five articles comparing the effectiveness of the Nd: YAG laser with that of the low-power diode laser (at low and high doses). Figure 1 presents a flowchart of the study selection process and the results of the literature search according to the PRISMA guidelines [19].

### 3.2. Main Results of the Studies

#### 3.2.1. Laser Nd: YAG

Table 1 presents the main features of the included studies that evaluate the effectiveness of the Nd: YAG laser in the treatment of dentinal hypersensitivity. In the studies, the treatment protocols for the Nd: YAG laser are:

- 1.5 W at 10 Hz and 100 mJ at 1064 nm for four sessions for a total of 60 s at 10 s intervals
- 1 W at 10 Hz for 60 s at 1064 nm for three sessions at 72 h intervals

In all these studies, the Nd: YAG laser produced excellent results in dentinal hypersensitivity treatment. In four of the studies (22-23-24-25), the Nd: YAG laser performed better than the desensitizer with which it was compared (Table 1).

#### 3.2.2. Low-Power Diode Laser

Table 2 presents the main features of the included studies that evaluate the effectiveness of the low-power diode laser in the treatment of dentinal hypersensitivity. Two protocols for the use of low-power diode lasers were found within the various studies:

1. Low-power low-dose diode laser:
  - 810 nm, 30 mW, and 10 J/cm^2^ for 9 s per point, with three sessions at 72 h intervals
  - 685 nm, 25 mW, and 9 Hz for 100 s for three sessions at 72 h intervals
2. Low-power, high-dose diode laser
  - 810 nm, 100 mW, and 40 J/cm^2^ for 11 s at one point on the cervical area and one in the apical area per point for three sessions at 72 h intervals
  - 810 nm, 0.5 W continuous-emission form; each tooth irradiated for 2 min in non-contact mode

All studies taken into consideration confirmed the validity of low-frequency diode laser treatment for dentinal hypersensitivity. Despite being compared with different desensitizing agents, the low-frequency diode laser has always produced better results and the association with the agents did not bring any improvement compared to the benefits obtainable with the laser alone [26,30], as shown in Table 2.

#### 3.2.3. High-Power Diode Laser

Table 3 shows the main features of the included studies that evaluate the effectiveness of the high-power diode laser in dentinal hypersensitivity treatment. In the studies, the protocols used for the high-frequency diode laser were:

- 3 W at 30 Hz and 980 nm for 20 s using a 300 µ fibre in pulsed mode
- 980 nm DL applied at 2 W in continuous-wave mode on the surface of the tooth to be treated, using a 320 µ fibre held perpendicular to the irradiated surface at a distance of 1 mm, each area irradiated twice for 20 s
- The teeth irradiated for 20 s with a beam of 0.2 W (980 nm, fibre 300 s, continuous-wave mode) and then for 20 s with 3 W DL output power in the first session; the teeth treated for 20 s with a 20 Hz and 0.2 W diode laser beam in the second and third sessions 48 and 96 h, respectively, after the initial visit

The results obtained were positive in terms of efficacy, even in the long term. The results of both studies [31,35] that used fluorine in combination with the high-power diode laser were better than those of studies using the fluorine-free protocols, whatever they were (Table 3).

#### 3.2.4. Nd: YAG Laser vs. Diode Laser

Table 4 presents the main features of the studies that compare and evaluate the Nd: YAG laser treatment and the diode laser treatment for dentinal hypersensitivity. The results obtained suggest that both lasers constitute an effective treatment. However, the Nd: YAG laser has provided greater benefits in dentinal hypersensitivity treatment than the low-power diode laser [36,37,38,39,40], as shown in Table 4.

## 4. Discussion

Dentinal hypersensitivity represents a dental disease of great clinical interest. The onset of painful symptoms can affect any dental element and patients of all ages, with a higher incidence in females aged 20 to 40 years [41,42]. There is a higher incidence of dentinal hypersensitivity in patients suffering from periodontal disease [43,44], with transient onset in patients undergoing scaling and root planing and periodontal surgery [45], and during dental whitening and conservative therapies [46].

Laser-assisted treatment of dentinal hypersensitivity appears to be effective in resolving immediate and long-term pain. Compared to conventional topical desensitizing agents, laser treatment, although more expensive, provides rapid results and is, therefore, faster for the patient [47].

Despite the previously described benefits of using the laser to treat hypersensitivity, the mechanisms by which the laser acts are still unclear and the cost–effectiveness ratio is low. Furthermore, at high temperatures, the potential thermal effects can damage sensitive pulp tissues [48].

Due to the small number of studies in the literature, a meta-analysis by Sgolastra et al. did not show these benefits in the laser treatment of hypersensitivity [49]. Hollande et al. suggested that further randomized double-blind clinical trials would be needed to evaluate the effectiveness of this technology [50]. Later, the data in the literature increased and, therefore, the goal of this review was to compare and evaluate the effectiveness of various protocols for the use of the Nd: YAG laser and the diode laser in treating dentinal hypersensitivity. The results show that both Nd: YAG and diode lasers (high and low power) effectively treat dentinal hypersensitivity. However, substantial differences have been highlighted in the results obtained with the different lasers or protocols used.

The Nd: YAG laser influences hypersensitivity by inducing occlusion or narrowing of the dentinal tubules and direct nerve analgesia [51]. Masumeh et al. showed that the Nd: YAG laser is significantly more effective than the diode laser and a dentin-bonding agent in reducing dentin hypersensitivity [52].

The Nd: YAG laser is costly. Therefore, it would be interesting to find out whether it can be replaced by the diode laser. The results obtained suggest that even the diode laser could be an excellent treatment in dentine hypersensitivity at both low and high power. Two included studies performed better using the high-power diode laser in combination with fluorine paints [31,35]; however, no benefit has been found when using the low-power diode laser in combination with other desensitizing agents [25].

According to a study by Lopes et al. [20], this treatment is more effective if Gluma Desensitizer is associated with the Nd: YAG laser. The articles analysed showed better results with this treatment than with desensitizers such as fluorine and sodium fluoride and compared to other lasers, such as Er: YAG [22,23,24,25].

A systematic review by Rezazadeh et al. [40] attempted to analyse all of the randomized clinical trials and comparative works, to evaluate the effectiveness of laser therapy in prevention and treatment of dentine sensitivity, and explained that previous research evaluating the desensitizing effect of lasers has used different approaches, which makes it difficult to compare their effectiveness, suggesting that the laser is an effective treatment for dentinal hypersensitivity. Some studies have not reported any significant difference between the laser and other desensitizing agents, and most studies have proposed better results (both rapid and long lasting) in combined modalities. The studies analysed have all obtained positive results in terms of the efficacy and validity of the laser treatment, both Nd: YAG and high- and low-power diode, considering them valid treatments for dentinal hypersensitivity. These results agree with those obtained in this study.

## 5. Conclusions

In conclusion, taking into consideration the limitations of this review, mainly due to the heterogeneity of the protocols adopted and the different desensitizing agents used in the studies, laser treatment, especially Nd: YAG laser treatment, seems to effectively address dentinal hypersensitivity. However, further studies, especially ones using diode lasers in association with fluorine-based agents, are necessary to establish their real effectiveness and evaluate which laser and protocol are the most suitable.

## Figures and Tables

**Table 1:** Characteristics of the included studies on the use of the Nd: YAG laser in dentinal hypersensitivity treatment.

Authors, Date, and Type of Study	Protocol	N° of Patients	N° of Teeth	Age Frames	Methods of Assessment	Follow-Up	Statistically Relevant	Conclusions
Lopes AO et al. (2013) [20] Randomized clinical study	1.5 W at 10 Hz and 100 mJ, 85 J/cm^2^; four irradiations performed, each for 15 s	24	33	---	VAS scale	6 months	Yes	The combination of Nd: YAG and Gluma Desensitizer is an effective treatment strategy that has immediate and lasting effects.
Bou Chebel F et al. (2018) [21] Randomized clinical study	1 W at 15 Hz for 60 s, twice	12	54	20–60 years	VAS scale	6 months	No	There was no significant difference between the two treatments, Nd: YAG laser and MI Varnish. Both treatments were effective and reduced dentin hypersensitivity immediately after treatment for up to 6 months.
Ciaramicoli MT et al. (2003) [22] Randomized clinical study	1 W at 10 Hz for 60 s at 1064 nm	20	145	23–63 years	Friedman Termal Test	6 months	Yes	The reduction in cervical dentinal hypersensitivity was statistically greater when the etiological factors were removed along with the application of the Nd: YAG laser.
Birang R et al. (2007) [23] Randomized clinical study	1 W at 15 Hz for 60 s, twice	9	63	---	VAS scale	6 months	Yes	The Nd: YAG laser is more effective than the Er: YAG laser in reducing pain in patients.
Talesara K et al. (2014) [24] Randomized clinical study	1 W at 10 Hz for 60 s, each element irradiated twice	20	80	25–65 years	VAS scale	6–9 months	Yes	The Nd: YAG laser was better when intra-group comparison was carried out at 9 months after treatment. Nd: YAG lasers are best in long-term treatment (up to 9 months) due to the dissolution of the dentinal tubules.
Hu C et al. (2004) [25] Randomized clinical study	1 W at 10 Hz for 60 s at 1064 nm	30	---	23–61 years	VAS scale	6 months	Yes	The Nd: YAG laser is safe and highly effective in the treatment of dentinal hypersensitivity.

**Table 2:** Characteristics of the included studies on the use of low-power diode lasers in dentinal hypersensitivity treatment.

Authors, Date, and Type of Study	Protocol	N° of Patients	N° of Teeth	Age Frames	Methods of Assessment	Follow-Up	Statistically Relevant	Conclusions
Bal MV et al. (2015) [26] Randomized clinical study	25 mW at 9 Hz for 100 s	21	154	20–60 years	VAS scale	6 months	Yes	Application of LLL or DP containing 8% arginine-calcium carbonate appears to be effective in decreasing DH. However, their combined use does not improve efficacy beyond what is achievable with either treatment alone.
Aranha AC et al. (2009) [27] Randomized clinical study	660 nm/3.8 J/cm^2^/15 mW	24	---	20–60 years	VAS scale	6 months	No	All therapies showed lower VAS sensitivity values than baseline, regardless of their different modes of action.
Jain A et al. (2020) [28] Randomized split-mouth clinical study	810 nm, 0.5 W continuous-emission form; each tooth irradiated for 2 min in non-contact mode	60	---	20–60 years	VAS scale	6 months	Yes	The diode laser is significantly more effective in dentinal hypersensitivity treatment for more than 6 months post treatment.
Flecha OD (2013) [29] Randomized double-blind clinical study	685 nm, 25 mW, and 9 Hz for 100 s for three sessions at 72 h intervals	62	434	---	Numeric rating scale	6 months	Yes	Cyanoacrylate is as effective as low-intensity laser in reducing DH. Furthermore, it is a more affordable procedure and can be used safely in DH treatment.
Lopes AO et al. (2015) [30] Randomized clinical study	Low-power low-dose diode: 30 mW, 10 J/cm^2^, 9 s per point, 810 nm, three sessions Low-power high-dose diode: 100 mW, 40 J/cm^2^, 11 s per point, 810 nm, three sessions	27	55	22–53 years	VAS scale	6 months	Yes	For the low-level lasers, distinct effects were observed for the different doses; however, both were effective in reducing pain for up to 6 months of clinical follow-up.

**Table 3:** Characteristics of the included studies on the use of high-power diode lasers in dentinal hypersensitivity treatment.

Authors, Data, and Type of Study	Protocol 1	Protocol 2	N° of Teeth	Age Frames	Methods of Assessment	Follow-Up	Statistically Relevant	Conclusions
Femiano F et al. (2013) [31] Randomized clinical study	980 nm DL applied at 2 W in continuous-wave mode on the surface of the tooth to be treated	Application of 5% NaF varnish + 980 nm DL at 2 W	262	21–64 years	VAS scale	6 months	Yes	Statistically significant improvements in the VAS scale were found at 1, 3, and 6 months in patients treated with high-power diode laser in combination with 5% NaF.
Raichur PS (2013) [32] Randomized clinical study	980 nm DL applied at 1 W		108	25–45 years	---	6 months	Yes	The 940 nm DL was not only effective but also resulted in better immediate relief than potassium fluoride and potassium nitrate gels in reducing DH.
Yilmaz HG (2011) [33] Randomized clinical study	980 nm DL applied at 1 W		244	18–58 years	VAS scale	6 months	Yes	Within the limitations of the study, GaAlAs laser irradiation was effective in treating DH and is a more comfortable and faster procedure than traditional DH treatment.
Tabibzadeh Z et al. (2018) [34] Randomized clinical study	3 W for 20 s at 980 nm and 30 Hz using a 300 µ fiber in pulsed mode once in the first group and three times in the second group		62	---	VAS scale	6 months	Yes	The use of both high-intensity and combined DL beams results in significantly reduced DH. There was no significant difference between combined and single-laser therapies in the treatment of tooth hypersensitivity.
Suri I et al. (2016) [35] Randomized clinical study	980 nm DL applied at 2 W in continuous-wave, non-contact mode using a 320 µ fiber radiated at 1 mm, each area irradiated twice for 20 s	Application of 5% NaF varnish + 980 nm DL at 2 W	---	20–59 years	VAS scale	6 months	Yes	Although all three groups showed improved DH reduction, 5% NaF paint with DL showed the best results among all groups.

**Table 4:** Characteristics of studies comparing Nd: YAG laser treatment and low-power diode laser treatment.

Authors, Date, and Type of Study	Protocol 1	Protocol 2	N° of Patients	N° of Teeth	Age Frames	Methods of Assessment	Follow-Up	Statistically Relevant	Conclusions
Lopes AO et al. (2017) [36] Randomized clinical study	Nd: YAG: 1.0 W, 10 Hz, 100 mJ, ≈85 J/cm^2^, 1064 nm	Low-power low-dose diode: 30 mW, 10 J/cm^2^, 9 s per point, 810 nm, three sessions Low-power high-dose diode: 100 mW, 40 J/cm^2^, 11 s per point, 810 nm, three sessions	32	117	22–53 years	VAS scale	18 months	No	After statistical analysis, all treatments were shown to be effective in reducing dentin hypersensitivity, and the results were considered not statistically different from those at 12 and 18 months.
Tabatabaei MH et al. (2018) [37] Randomized clinical study	Nd: YAG: 1.0 W, 10 Hz, 100 mJ, ≈85 J/cm^2^, 1064 nm	810 nm at 30 mW for 9 s per point	22	135	25–58 years	VAS scale	6 months	Yes	The efficacy of the Nd: YAG laser in reducing dentin hypersensitivity was significantly superior to that of other modalities at 3 and 6 months.
Dilsiz A et al. (2010) [38] Randomized clinical study	Nd: YAG: 1064 nm, 100 mJ/pulsed mode, 15 Hz, 100 s	Laser diode: 808 nm at 100 mW for 20 s	24	96	18–52 years	VAS scale	3 months	Yes	Er: YAG, Nd: YAG, and diode lasers can be used to reduce DH. Nd: YAG laser irradiation is more effective in treating DH than Er: YAG and diode laser. Within the limitations of the study, the Nd: YAG laser appeared to be a suitable tool for successful DH reduction, especially since the 3 month results of this treatment modality are promising.
Dilsiz A et al. (2009) [39] Randomized clinical study	Nd: YAG: 1 W and 10 Hz for 60 s at 1064 nm	Laser diode: 685 nm at 25 mW and 9 Hz for 100 s	14	56	19–51 years	VAS scale	3 months	Yes	Desensitivity of teeth with gingival recession was more effective with the Nd: YAG laser than with the diode laser. The Nd: YAG laser appears to be a promising new tool for successfully reducing DH.
Rezazadeh F et al. (2019) [40] Review of the literature			32	117	22–53 years	VAS scale	---	Yes	Among the various types of lasers, the application of the Nd: YAG laser has shown the best results in the treatment of dentinal hypersensitivity.
